# Efficacy of the Flow Re-direction Endoluminal Device for cerebral aneurysms and causes of failed deployment

**DOI:** 10.1007/s00234-021-02858-w

**Published:** 2021-11-13

**Authors:** Kenichiro Suyama, Ichiro Nakahara, Shoji Matsumoto, Yoshio Suyama, Jun Morioka, Akiko Hasebe, Jun Tanabe, Sadayoshi Watanabe, Kiyonori Kuwahara

**Affiliations:** Department of Comprehensive Strokology, Fujita Health University School of Medicine, 1-98 Dengakugakubo, Kutsukake-cho, Toyoake, Aichi Japan

**Keywords:** FRED, Deployment failure, Efficacy, Cerebral aneurysm, Flow diverter

## Abstract

**Purpose:**

The Flow Re-direction Endoluminal Device (FRED) has recently become available for flow diversion in Japan. We have encountered cases that failed to deploy the FRED. In this study, we report our initial experience with the FRED for cerebral aneurysms and clarify the causes of failed FRED deployment.

**Methods:**

A retrospective data analysis was performed to identify patients treated with the FRED between June 2020 and March 2021. Follow-up digital subtraction angiography was performed at 3 and 6 months and assessed using the O’Kelly-Marotta (OKM) grading scale.

**Results:**

Thirty-nine aneurysms in 36 patients (average age: 54.4 years) were treated with the FRED. The average sizes of the dome and neck were 9.9 mm and 5.2 mm, respectively. In nine patients, additional coiling was performed. In one patient (2.6%), proximal vessel injury caused direct carotid-cavernous fistula during deployment. Ischaemic complications were encountered in one patient (2.6%) with transient symptoms. Angiographic follow-up at 6 months revealed OKM grade C or D in 86.6% of patients. FRED deployment was successful in 35 (92.1%) procedures. In the failure group, the differences between the FRED and the minimum vessel diameter (*P* = 0.04) and the rate of the parent vessel having an S-shaped curve (*P* = 0.04) were greater than those in the success group.

**Conclusions:**

Flow diversion using the FRED is effective and safe for treating cerebral aneurysms. The use of the FRED for patients with an S-shaped curve in the parent vessel and oversizing of more than 2 mm should be considered carefully.

## Introduction

The Flow Re-direction Endoluminal Device (FRED; MicroVention-Terumo, Tustin, CA, USA) is a flow diverter (FD) device characterised by a dual-layer self-expanding structure. The FRED has been used for flow diversion in Europe and several other countries and has recently become available in Japan. The FRED is indicated for use for the petrous segment of the internal carotid artery (ICA) to the A1 region of the anterior cerebral artery or M1 region of the middle cerebral artery (MCA) and the intracranial vertebral and basilar arteries for endovascular treatment with wide-necked (neck width ≥ 4 mm or dome-to-neck ratio < 2) saccular or fusiform intracranial unruptured aneurysms. High safety and efficacy have been reported in multi-institutional studies [1–4], but stent deployment has failed in some cases.

In this study, we report our initial experience of the FRED with short-term results in the real world and clarify the causes of failed deployment.

## Materials and methods

This single-centre retrospective study evaluated the angiographic and clinical data of consecutive patients treated with the FRED for cerebral unruptured aneurysms from June 2020 to May 2021. Our treatment indications with the FRED were as follows: (1) the maximum dome diameter was > 5 mm and (2) wide-necked (neck width 4 mm or dome-to-neck ratio < 2) saccular or fusiform intracranial unruptured aneurysms. The final decision regarding whether to perform a surgical treatment was made in a comprehensive conference including neurosurgeons, neurointerventionists and neurologists. We avoided treating patients with prominent parent vessel stenosis, which made deploying a FD difficult. The following data were obtained and retrospectively reviewed from medical charts. Baseline characteristics, including demographics, medical history and aneurysm characteristics, were recorded. The treatment characteristics, including the number and size of the FREDs, parent vessels, procedure time, adjunctive techniques and technical problems, were collected. To clarify the cause of failed deployment, we compared groups based on the success or failure of FRED deployment. All intraprocedural, periprocedural and delayed complications were reported. Clinical outcomes were evaluated based on the modified Rankin Scale (mRS) [5] at discharge. The mRS was evaluated by a neurologist at admission and discharge.

The institutional ethics committee approved this study (approval number: HM 20–572). The need for written informed consent was waived with the opportunity to opt out posted on the institutional website because of the retrospective nature of the study, which included an analysis of routine programmatic data.

### Perioperative management

Before the procedure, all patients received dual antiplatelet therapy with clopidogrel (CPG) at 75 mg and aspirin at 100 mg for 14 days. P2Y12 reaction unit (PRU) was monitored by VerifyNow (Accumetrics, San Diego, CA, USA) 2 days before the procedure, and CPG was changed to prasugrel (PSG) in all patients [6]. In patients whose PRU was greater than 210, a loading dose of PSG at 20 mg was administered; subsequently, a dose of PSG at 3.75 mg per day was maintained. In patients whose PRU was between 60 and 210, a dose of PSG (3.75 mg) per day was administered. In patients whose PRU was less than 60, a dose of PSG (1.9 mg) per day was administered. PRU was re-examined 4 days after the procedure to check for PSG effectiveness. Dual antiplatelet therapy was continued for at least 6 months after the procedure. The institutional off-label use committee approved the off-label use of PSG.

### Endovascular treatment

All procedures were performed under general anaesthesia with systemic heparinisation, aiming to maintain an activated clotting time of > 250 s. The transfemoral approach was used for all patients. An 8F guiding catheter (ROADMASTER, GOODMAN, Aichi, Japan) was inserted into the ICA or vertebral artery as appropriate. A 5F distal access-guiding catheter (SOFIA SELECT, MicroVention-Terumo) was placed as close as possible to the aneurysm through the guiding catheter. The FRED was deployed via a HeadwayPlus27 (MicroVention-Terumo) through the distal access-guiding catheter. In an aneurysm with a size greater than 15 mm and without intra-aneurysmal thrombosis, treatment was performed with additional coils. Coils were also used for aneurysms with irregular shapes, including blebs, even in cases of less than 15 mm in diameter. The amount of coil used covered the whole wall of the aneurysm. When coil embolisation was performed, a 6F SOFIA SELECT was used as a distal access-guiding catheter. Headway Duo (MicroVention-Terumo) or Phenom17 (Medtronic, Irvine, CA, USA) was placed in the aneurysm in parallel with Headway 27 through 6F SOFIA SELECT. Coils were deployed through the HeadwayDuo or Phenom17 using the jailing technique. Partial coil embolisation was preceded by the simple or balloon-assisted technique when the coils stay stable before FRED deployment. The FRED size was selected based on the maximum diameter of the parent vessel. After FRED deployment, high-resolution cone-beam computed tomography (CT) was performed to evaluate the wall apposition of the FRED. Postdilatation with a balloon catheter was performed using a compliant balloon for incomplete apposition.

### Follow-up protocol

Angiographic follow-up was performed at 3 and 6 months after the procedure, and occlusion rate and in-stent stenosis were assessed. The occlusion rate was evaluated using the O’Kelly-Marotta (OKM) grading scale [7]. OKM grading scales C and D were defined as adequate occlusion. Patients with technical failure to deploy the FRED were excluded from angiographic follow-up but were included in the clinical follow-up. All angiograms were independently evaluated in random order by two neurointerventionists. All evaluators had more than 10 years of experience. In case of disagreement, a consensus was reached between the two interventionists.

### Statistical analyses

Data are presented as mean ± standard deviation or median and interquartile range for continuous variables and frequencies for categorical variables. Statistical analysis was performed using Student’s *t*-test, Mann–Whitney *U* test or Fisher’s exact test. Statistical significance was set at *P* < 0.05. Statistical analyses were performed using EZR software.

## Results

During the study period, 39 aneurysms in 36 patients were treated with the FRED at our institute. The characteristics of the patients and aneurysms are presented in Table 1. Eight of the 39 aneurysms (20.5%) were recurrent aneurysms after previous treatment. Five recurred after endovascular treatment (coil embolisation, 4; stent-assisted coil embolisation, 1) and two after clipping. Regarding aneurysm location, 23 (59.0%) aneurysms were located at the ICA (ICA cavernous, 2; ICA paraclinoid, 18; ICA anterior choroidal, 3), 3 (7.7%) at the MCA (MCA bifurcation, 2; M1 segment, 1), 11 (28.2%) at the vertebral artery, and 2 (5.1%) at the basilar artery. The mean aneurysm size was 9.9 mm, and the mean neck size of the saccular aneurysms was 5.2 mm. Eight (20.5%) aneurysms were large (≤ 10 mm, < 25 mm) and four (10.2%) were giant (≤ 25 mm).

**Table 1:** Baseline characteristics

Characteristics	Value
No. of aneurysms	39
No. of patients	36
No. of procedures	38
Age, years	54.4 ± 15.2
Male	8 (22.2%)
Symptomatic	1 (2.8%)
Aneurysm size, mm	9.9 ± 6.7
Neck, mm (saccular)	5.2 ± 2.8
Dome/neck ratio (saccular)	1.7 ± 0.9
Aneurysm morphology	
Saccular	26 (66.6%)
Fusiform/dissecting	13 (33.3%)
Location	
ICA cavernous	2 (5.1%)
ICA paraclinoid	18 (46.2%)
ICA AChA	3 (7.7%)
MCA	3 (7.7%)
VA	11 (28.2%)
BA	2 (5.1%)
Recurrence after previous treatment	8 (20.5%)

*mRS*, modified Rankin scale; *ICA*, internal carotid artery; *AChA*, anterior choroidal artery; *MCA*, middle cerebral artery; *VA*, vertebral artery; *BA*, basilar artery

**Fig. 1:**
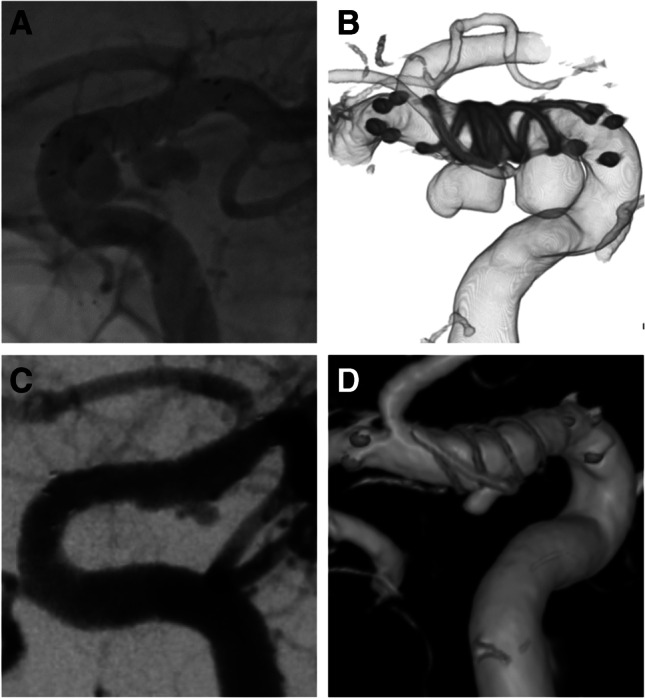
Images from a case involving a 55-year-old woman with ICA paraclinoid and anterior choroidal aneurysms. Digital subtraction angiography (**A**) and three-dimensional image (**B**) just after the treatment with FRED. Digital subtraction angiography (**C**) and three-dimensional image (**D**) after 6 months show complete occlusion of the paraclinoid aneurysm (OKM grading scale D) and entry remnant of the anterior choroidal aneurysm (OKM grading scale C). *OKM*, O’Kelly-Marotta

Three patients had an additional aneurysm, and two of them had one aneurysm each at the right and left ICA, and they were treated with different FREDs in the same session. Another patient had two aneurysms near the left ICA that were treated using the same FRED (Fig. 1). In one patient, two FREDs were used for one aneurysm because the effective length of the FRED did not completely cover the aneurysm neck.

**Fig. 2:**
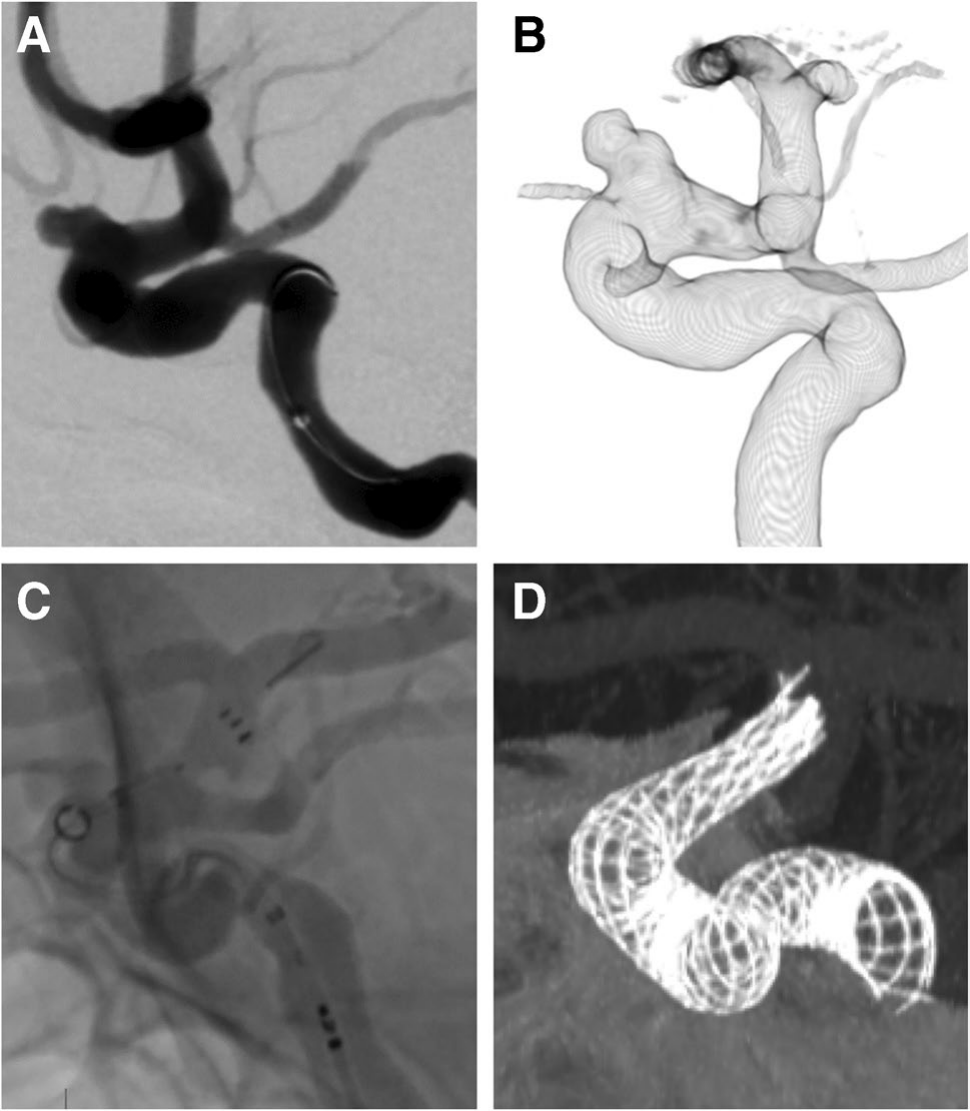
Images from a case involving a 45-year-old woman with paraophthalmic ICA aneurysm. Digital subtraction angiography (**A**) and three-dimensional image (**B**). FRED was not expanded and retrieved (**C**). The wall apposition of the PED was confirmed by cone-beam computed tomography with diluted contrast (**D**). *ICA*, internal carotid artery; *FRED*, Flow Re-direction Endoluminal Device; *PED*, pipeline embolisation device

The results of this procedure are shown in Table 2. Deployment was successful in 35 of 38 procedures (92.1%), whereas the FRED was not opened in 3 (7.9%) procedures (Fig. 2). All three patients who failed to deploy the FRED had an ICA aneurysm. In two of three patients, the FRED was converted to the Pipeline embolisation device (PED; Medtronic), and the PED was deployed successfully. In another patient with an ICA cavernous aneurysm, the initial unopened FRED was retrieved. Headway 27 was navigated up to the M1 portion of the MCA, and another FRED was introduced, which started to open the initial flare at the ICA top and was pulled down to deploy the FRED at an adequate position.

**Table 2:** Results of procedure

	**Value**
Technical success	35 (92.1%)
Procedure time, min	72 (60–112)
Adjunctive techniques	
Coil embolisation	9 (23.1%)
Postdilatation with balloon	23 (59.0%)
Complications	
Intra-/periprocedural	
Ischaemic	1 (2.6%)
Haemorrhagic	1 (2.6%)
Delayed	
Ischaemic	0 (0%)
Haemorrhagic	0 (0%)
Morbidity	0 (0%)
Mortality	0 (0%)

**Fig. 3:**
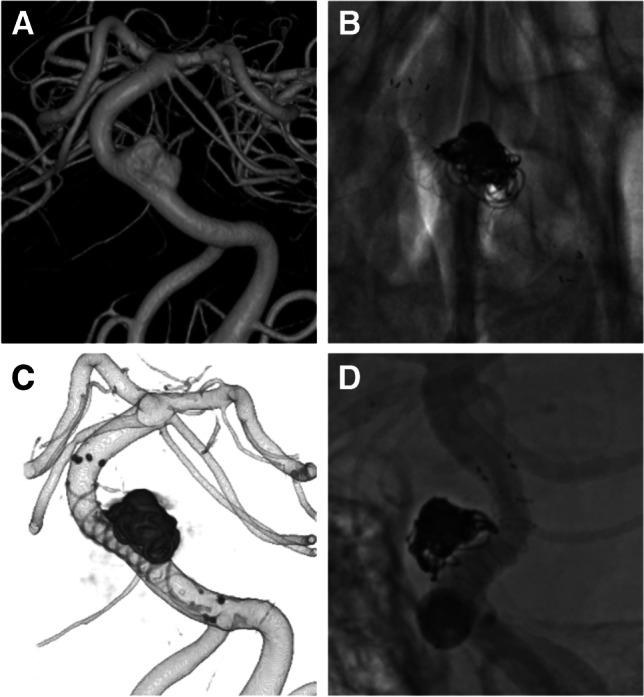
Images from a case involving an 80-year-old woman with basilar dissecting aneurysm. Three-dimensional (3D) image pre-treatment (**A**). Digital subtraction angiography (**B**) and 3D image (**C**) just after the treatment with FRED additional coiling. Follow-up angiography after 6 months (**D**) shows complete occlusion of the aneurysm (OKM grading scale D). *FRED*, Flow Re-direction Endoluminal Device; *OKM*, O’Kelly-Marotta

In nine aneurysms, treatment was performed with additional coiling (Fig. 3). Postdilatation with a balloon was performed in 23 aneurysms (59.0%), and all patients in which balloon angioplasty was performed were confirmed to gain sufficient apposition to the parent vessel by high-resolution cone-beam CT. Intraprocedural and periprocedural complications were observed in two patients (5.2%). In one of the patients with failed deployment, proximal vessel injury caused asymptomatic direct carotid-cavernous fistula (CCF) during deployment. The fistulous point was covered with two PEDs, as described above and the shunt flow gradually decreased in the follow-up digital subtraction angiography (DSA). Ischaemic infarction occurred in one patient (2.6%) with transient paralysis of the arm during the perioperative period. No permanent morbidity or mortality was observed. The post-procedural PRU with the administration of PSG was significantly decreased compared to pre-treatment PRU with the administration of CPG (146 (108.8–178.3) vs 207 (153.0–225.5), *P* < 0.01).

Angiographic follow-up at 3 months and 6 months were performed in 27 and 15 aneurysms, respectively. The results of angiographic follow-up are shown in Table 3. Angiographic follow-up at 6 months revealed OKM grade D in 10 (66.7%). The rate of adequate occlusion (OKM grade C or D) at 6 months was 86.6%. In-stent stenosis of greater than 50% was recorded in one patient. No jailed branch with the FRED was occluded, and no ischaemic complications were encountered during the follow-up period.

**Table 3:** Status of obliteration by angiographic follow-up at 3 and 6 months

OKM grading scale	3 months (*n* = 27)	6 months (*n* = 15)
Grade A	2 (7.4)	0 (0)
Grade B	4 (14.8)	1 (6.7)
Grade C	8 (29.6)	4 (26.7)
Grade D	13 (48.1)	10 (66.7)

*OKM*, O’Kelly-Marotta

**Fig. 4:**
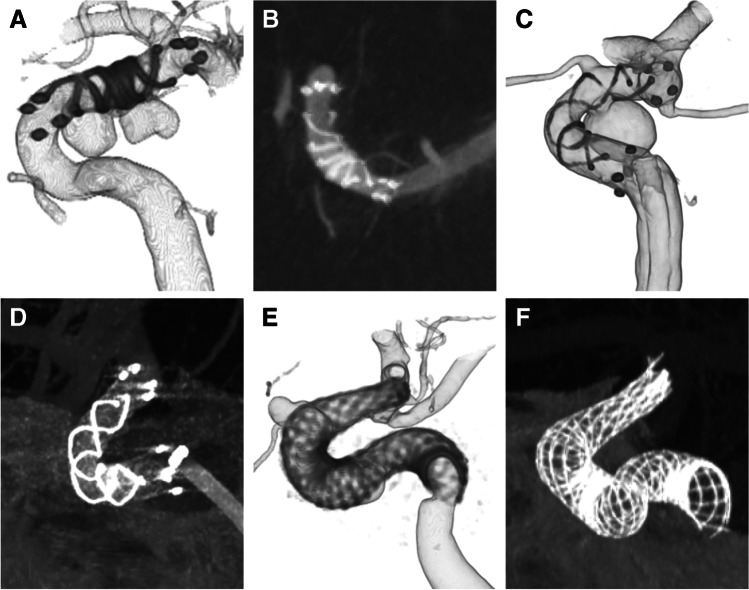
Images of the parent vessel curve detaining FRED or PED. **A**, **B** The curve of ICA C1–C2 portion and include a loose one curve. **C**, **D** The curve of carotid siphon and include one curve. **E**, **F** The parent vessel, including more than two curves and formed an S-shaped curve. *ICA*, internal carotid artery; *FRED*, Flow Re-direction Endoluminal Device; *PED*, pipeline embolisation device; *ICA*, internal carotid artery

A comparison of the clinical and angiographic features between the failure and success groups in FRED deployment is shown in Table 4. In the failure group, the differences between the FRED and minimum parent vessel diameter were significantly greater than that in the success group (failure vs success group, 2.2 vs 1.0 mm, *P* = 0.04). Furthermore, the rate of parent artery curvature in which the FRED was deployed containing more than two curves (S-shaped curve) was significantly higher in the failure than success group (66.7% vs 5.6%, *P* = 0.02) (Fig. 4). The procedure time was significantly longer in the failure than success group (141 min vs 72 min, *P* = 0.02).

**Table 4:** Comparison of the characteristics between the failure group and the success group in deployment

	Success (*n* = 36)	Failure (*n* = 3)	*P* value
Age, years	52 (47–62 years)	68 (57–74 years)	0.33
Male	8 (22.2)	0 (0)	1.0
Aneurysm size, mm	7.6 (6.0–11.2)	6.5 (5.9–9.0)	0.62
Oversizing, mm	1.0 (0.8–1.4)	2.2 (1.7–2.4)	0.04
S-shaped curve	2 (5.6)	2 (66.7)	0.02
Posterior circulation	13 (36.1)	0 (0)	0.54
Procedure time, min	72 (58–100)	141 (136–154)	0.02

*Oversizing*, the differences between FRED and minimum parent vessel diameter detaining FRED; *S-shaped curve*, patients whose parent vessel detaining FRED included more than two curves

## Discussion

The FRED has been used in Europe since 2012, and several multicentre studies have reported its high safety and efficacy. Previous studies have shown that the morbidity and mortality rates were 1.8–1.3% and 1.6–1.3%, respectively [1–4].

In this study, symptomatic ischaemic complications were recorded in only one patient (2.6%) with transient symptoms. The rate of ischaemic complications was lower than those in previous reports relating to FD [1–4, 8–11]. One of the reasons for the low ischaemic complication rate may be our antiplatelet therapy regimen using PSG. The FRED has high metal coverage. In some cases, the FRED is deployed in a smaller vessel such as the MCA. Therefore, strong antiplatelet therapy using PSG may be effective in preventing ischaemic complications. Dual antiplatelet therapy is standard in the perioperative periods of FD. Still, some individuals show genetic variation, failing to respond to CPG [12–15], and there is no consensus regarding the exact types and doses of antiplatelet therapy. PSG has been reported to be effective for patients with inadequate response to CPG [6]. In this study, all patients received PSG and the PRU was significantly decreased after changing CPG to PSG. The use of PSG may be the main reason for low ischaemic complications. A haemorrhagic complication was recorded in one patient, and this was the proximal vessel injury during the FRED deployment. Therefore, PSG was less likely to be related. Our sample size was small, and further studies are necessary to validate the efficacy and safety of our antiplatelet therapy regimen.

Delayed complications of treatment using FD include delayed aneurysmal rupture and delayed haemorrhage (intraparenchymal haemorrhage and subarachnoid haemorrhage). Delayed aneurysmal rupture is observed in 3% of cases, and giant and symptomatic aneurysms are risk factors for delayed rupture [1–4, 9–11]. Delayed haemorrhage is observed in 3–4% of cases [9–11]. One of the causes of delayed haemorrhage is embolisation of coating materials of interventional devices to small distal vessels [16, 17]; however, there is no consensus on the cause of delayed haemorrhage. In this study, delayed complications were not observed. The small proportion of large and giant aneurysms and the strategy of adding coils in cases of aneurysms greater than 15 mm might be related to the low delayed complication rate.

Previous studies have reported a complete occlusion (OKM grade D) rate of 61–82% and adequate occlusion (OKM grade C or D) rates of 69.5–94.0% at 3–6 months after treatment [1–4]. In a previous study, angiographic follow-up was performed at 3–6 months after treatment, and there were ranges for each study [1–4]. In this study, angiographic follow-up was performed at dense intervals 3 and 6 months after treatment. Adequate occlusion was observed at 3 months in 21 of the 27 aneurysms (77.8%). This result shows that treatment efficacy was obtained in a relatively early phase after treatment. The complete occlusion rate was slightly lower than those in previous studies [1–4]. The occlusion rate may increase in the follow-up period.

The side branch occlusion rate has been reported to be 1.4% at the 6-month follow-up [18] and is associated with the placement of multiple overlapping FDs [19]. In this study, no side branch occlusion was observed during the perioperative and follow-up periods. The FRED does not require postdilatation with a balloon. However, in this study, more than half of the patients underwent postdilatation with a balloon-based on cone-beam CT to maintain good wall apposition. Good wall apposition with balloon dilatation may be associated with the absence of side branch occlusion.

Previous studies have reported that the success rate of deployment was 95.1–98.3% [1–4]. A high success rate has been reported, but there were some cases in which stent deployment failed. In this study, we found the characteristics of the deployment failure case.

The success rate of deployment was 92.1% in the present study, which was slightly lower than those in previous reports. The FRED did not expand at vessel tortuosity in patients with failed deployment, and deployment was not possible. The FRED size was selected based on the maximum diameter of the parent vessel. In the failure group, the differences between FRED and minimum parent vessel diameter in detaining the FRED were significantly greater than those in the success group. In braided stents, oversizing of the parent vessel causes lying down of the stent struts, which reduces the expansion force of the stent. Kocer et al. have reported that distal oversizing of greater than 1 mm might cause opening problems at tight curves [20]. In our study, deployment was possible in cases where oversizing was 1 mm, but deployment was not possible in cases where oversizing was greater than 2 mm. Additionally, in two of three patients with failed deployment, their parent vessels detaining the FRED had more than two curves and formed an S-shaped curve. The success rate of FRED deployment was low in the S-shaped parent vessel. When the parent vessel has an S-shaped curve (more than two curves), the curves do not exist on the same plane surface; therefore, distortion of the FRED occurs, causing poor expansion of the FRED. Oversizing the parent vessel extends the total length of the FRED and leads to the S-shaped formation of the FRED. It is essential to select the optimal length and size of the FRED to avoid S shaping and prevent distortion of the FRED. According to our results, oversizing of greater than 2 mm and an S-shaped curve caused failed deployment at the vessel tortuosity; the FRED should be used carefully in such cases. To the best of our knowledge, this fact has not yet been reported in earlier literature. We have started the next project of the bench test using three-dimensional vessel models to elucidate this unopening phenomenon and examine the performance tests of FRED and PED.

We would like to report the results of this project in the next paper.

In two of three patients in whom the FRED failed to deploy, the FRED was converted to a PED, and the PED was deployed successfully. The PED can be expanded in the tortuous vessel by system push and system pull with effort. Conversely, when the FRED was not expanded, the system push and system pull did not help to expand the FRED as with the PED.

In our institute, the FRED was selected to treat cerebral aneurysms as the first priority for this period. However, we now select the PED in cases in which the parent vessel forms an S-shaped curve and oversizing is greater than 2 mm.

Our study has several limitations, including its single-centre retrospective design and relatively small sample size. Additionally, the follow-up period was short, and ischaemic complications and the status of obliteration may change. Further follow-up and prospective studies are necessary to validate our findings.

## Conclusion

Flow diversion using the FRED appears to be effective and safe for treating cerebral aneurysms. The use of the FRED for patients with two curves in the parent vessel and oversizing greater than 2 mm should be considered carefully.

## Data Availability

Data are available from the authors with the permission of a third party. The data that support the findings of this study are available from the corresponding author, upon reasonable request.
